# Safety and efficacy of intravesical instillation of botulinum toxin-A in the treatment of interstitial cystitis/bladder pain syndrome and overactive bladder: a systematic review and meta-analysis

**DOI:** 10.3389/fphar.2025.1586845

**Published:** 2025-04-15

**Authors:** Yongheng Zhou, Zitian He, Tianyu Xiang, Xiaoyan Cao, Huiling Cong, Qinggang Liu, Haoyu Sun, Limin Liao

**Affiliations:** ^1^ Department of Urology, China Rehabilitation Science Institute, Beijing, China; ^2^ Department of Urology, Qilu hospital of Shandong University, Jinan, Shandong, China; ^3^ Department of Urology, University of Health and Rehabilitation Sciences, Qingdao, Shandong, China; ^4^ Department of Urology, China Rehabilitation Research Center, Beijing, China; ^5^ Beijing Key Laboratory of Neural Injury and Rehabilitation, Beijing, China; ^6^ School of Rehabilitation, Capital Medical University, Beijing, China; ^7^ Department of Rehabilitation, Yuying Children’s Hospital, the Second Affiliated Hospital of Wenzhou Medical University, Wenzhou, Zhejiang, China; ^8^ Department of Quality Management, The First Medical Center of PLA General Hospital, Beijing, China; ^9^ Department of Urology, Qingdao Municipal Hospital, Qingdao, China

**Keywords:** botulinum toxin A, intravesical instillation, OAB, IC/BPS, meta-analysis

## Abstract

**Background:**

The effectiveness and safety of the instillation of botulinum toxin A (BTX-A) remain subjects of controversy. The meta-analysis was performed to assess the efficacy and safety of a novel intravesical instillation of BTX-A for managing overactive bladder (OAB) or interstitial cystitis/bladder pain syndrome (IC/BPS).

**Method:**

The randomized controlled trials were retrieved from PubMed, EMBASE, and the Cochrane Library databases up to 29 January 2024. The studies included in the analysis focused on the intravesical instillation of BTX-A in patients with OAB or IC/BPS. The data extraction was independently conducted by two reviewers. The random effects model was utilized for the assessment in order to compute the overall effect sizes. The heterogeneity tests and subgroup analyses were conducted.

**Results:**

The meta-analysis and subgroup analysis did not reveal any statistically significant differences. However, the results of the meta-analysis indicated that intravesical instillation of BTX-A could reduce episodes of urgency urinary incontinence (UUI) (overall weighted mean difference [WMD] = −0.85; 95% confidence interval [CI]: -2.99 to 1.29). In subgroup analysis, an increase in void volume (VV) at 4 weeks of follow-up (WMD = −31.99; 95% CI: -70.53 to 6.54) was observed compared to that at 12 weeks (WMD = −1.73; 95% CI: -16.98 to 13.53). In contrast to the groups receiving more than 200 units of BTX-A, patients in the group receiving 200 units or fewer of BTX-A (WMD = −16.89; 95% CI: -41.14 to 7.35) exhibited a significantly greater increase in VV.

**Conslusion:**

The intravesical instillation of BTX-A appears to be a viable administration route that may reduce UUI and VV to some extent. In terms of safety, intravesical instillation of BTX-A demonstrated a reduced risk of UTI and post-void residual compared to the placebo group.

**Systematic Review registration:**

https://www.crd.york.ac.uk/PROSPERO/view/CRD42024517877.

## Introduction

Overactive bladder (OAB) and interstitial cystitis/bladder pain syndromes (IC/BPS) are prevalent chronic disorders of the lower urinary tract characterized by dysfunction, with complex and often unknown etiologies. These conditions significantly impair patients’ quality of life and psychological wellbeing, while also imposing a substantial economic burden on families and the healthcare system (Milsom et al., 2014; Doggweiler et al., 2017). Among these disorders, OAB is defined by urinary urgency and frequency, whereas IC/BPS is primarily characterized by chronic pelvic pain associated with the bladder, often accompanied by urinary frequency, urgency, and nocturia (Hanno and Dmochowski, 2009; Doggweiler et al., 2017; Robinson and Cardozo, 2019).

Behavior modification and pharmacotherapy are frequently utilized in clinical settings as conservative treatment options for OAB and IC/BPS. Nevertheless, the prolonged use of oral medications such as antimuscarinic cholinergics, β3 agonists, and α-blockers may lead to various side effects, including dry mouth, constipation, blurred vision, and compromised cognitive function. These side effects significantly diminish patient compliance with the treatment regimen (Yeowell et al., 2018; Gerlach et al., 2022; Dmochowski et al., 2023).

Botulinum toxin (BTX) is a potent neurotoxin protein that can block the release of acetylcholine from nerve fibers, resulting in muscle paralysis (Simpson, 1980). Furthermore, BTX can inhibit the release of neurotransmitters like substance P, adenosine triphosphate (ATP), and calcitonin gene-related peptide, among others. This action leads to the production of analgesic and anti-inflammatory effects (Aoki, 2003; Kaya et al., 2013). BTX is classified into types A-G. Botulinum toxin type A (BTX-A), recognized for its prolonged duration of action, has been utilized in the management of lower urinary tract disorders for more than 3 decades. BTX, with a high molecular weight of 150 kDa, cannot permeate the epithelial barrier of the urinary tract before exerting its effects on the submucosal nerve plexus (Kuo, 2022). In 1988, injections of BTX-A into the urinary sphincter were employed to alleviate detrusor-sphincter dyssynergia in patients with spinal cord injury (Dykstra et al., 1988). By 2015, the American Urological Association guidelines had recommended this technique as the standard therapy for drug-refractory OAB and IC/BPS (Hanno and Dmochowski, 2009; Gormley et al., 2015). Numerous clinical studies have documented the effectiveness of intravesical BTX-A injections in managing OAB and IC/BPS (Giannantoni et al., 2019; De Rienzo et al., 2022; Liao et al., 2022; Delaval et al., 2023).

However, adverse events have also been reported, including drug leakage, hematuria, urinary tract infections (UTIs), urinary retention, and an increase in post-void residual volume (PVR) (Tyagi et al., 2006). Therefore, the development of a novel noninvasive method for delivering BTX-A to the bladder represents a promising avenue for future research. Several clinical randomized controlled trials have reported the effectiveness of a novel noninvasive intravesical instillation of BTX-A for OAB or IC/BPS (Chuang et al., 2014; Kuo et al., 2014; Krhut et al., 2016; Chuang and Kuo, 2017; Chermansky et al., 2022). However, the findings remain contentious. Recent updated evidence indicates that the efficacy of bladder instillation of BTX-A using hydrogel as a drug delivery mechanism has not demonstrated significant results (Chermansky et al., 2022).

Consequently, the effectiveness of novel carriers for BTX-A in bladder instillation for OAB or IC/BPS, as well as the changes in both near-term and long-term efficacy and voiding parameters, remains inconsistent across different studies. Therefore, a meta-analysis is necessary to clarify the changes in efficacy.

## Material and methods

The meta-analysis and systematic review were conducted in compliance with Meta-Analyses (PRISMA) statement (Liberati et al., 2009) and registered on the International Prospective Register of Systematic Reviews website (https://www.crd.york.ac.uk/prospero/) with registration number CRD42024517877.

### Databases and search methods

The three databases used for literature search were PubMed, EMBASE, and the Cochrane Library. All English publications were searched up to 29 January 2024, according to the Medical Subject Headings (MeSH) terms, which included “Cystitis, Interstitial,” “Urinary Bladder, Overactive,” “Botulinum Toxins, Type A,” and equivalent free-text terms in PubMed. YH Z and ZT H independently conducted the searches and screenings, followed by a cross-check of their findings. It is important to note that references were also searched to avoid missing relevant articles. Any disagreements that arise during this process must be discussed and resolved.

### The article inclusion and exclusion criteria

Inclusion criteria: 1. The type of article is limited to RCTs; 2. The article must be highly relevant to intravesical botulinum toxin instillation for OAB and IC/BPS patients; 3. Major endpoints include: frequency of episodes, episodes of urinary urgency, urgency urinary incontinence (UUI), void volume (VV), nocturia, overactive bladder syndrome score (OABSS), patient perception of bladder condition (PPBC), and cystometric capacity. Minor endpoints include maximum flow rate (Q_max_) and UTI events. At least one of the above indicators was reported.

Exclusion criteria: 1. No human trials; 2. Irrelevant to the topic; 3. Full text was not available.

### Data extraction

The basic data extraction included the following elements: authors, year of publication, and country, study period, study design type, diagnostic, sample size, age, gender, interventions agents, and follow-up time.

ZT H and YH Z extracted the relevant data from the articles that met the inclusion criteria and compiled it into a table (Table 1). Subsequently, TY X proofread the results. All uncertainties were addressed through discussion.

**TABLE 1:** Baseline characteristics of included studies.

No.	Author, year	Country	Period	Diagnostic	Study type	Sample	Females/Males	Patients’ age		Experimental agent	Control agent	No. of sample Tx/Pb	Follow-up (m)
								Tx	Pb				
1	Kuo et al. (2014)	Taiwan, China	NA	OAB	RCT	24	14/10	67 (38–82)		Lipotoxin (200U BTX-A+80 mg liposomes)	normal saline	12/12	1,3
2	^1^ Chuang et al., 2014	Taiwan, China	2011–2013	OAB	RCT	62	33/29	64 no SD	66 no SD	Lipotoxin (200U BTX-A+80 mg liposomes)	normal saline	31/31	1
2^*^	^2^ Chuang et al., 2014	Taiwan, China	2011–2013	OAB	RCT	23	NA	NA	NA	Lipotoxin (200U BTX-A+80 mg liposomes)	normal saline	12/11	3
3	^1^ Krhut et al., 2016	Czech Republic	NA	OAB	RCT	20	20/0	52.3 ± 8.4	52.2 ± 9.1	50 mL TC-3 gel+200U BTX-A	normal saline	9/11	1
3^*^	^2^ Krhut et al., 2016	Czech Republic	NA	OAB	RCT	21	21/0	55.3 ± 11.9	52.2 ± 9.1	50 mL TC-3 gel+200U BTX-A+25% DMSO	normal saline	10/11	1
4	^1^ Chuang and Kuo, 2017	Taiwan, China	NA	IC/BPS	RCT	62	54/8	53.9 ± 12.9	55.9 ± 8.6	Lipotoxin (200U BTX-A+80 mg sphingomyelin)	normal saline	31/31	1
4^*^	^2^ Chuang and Kuo, 2017	Taiwan, China	NA	IC/BPS	RCT	59	53/6	47.8 ± 9.9	55.9 ± 8.6	200U BTX-A + normal saline	normal saline	28/31	1
5	^1^ Chermansky et al., 2022	United States of America	2017–2020	OAB	RCT	294	106/9	60.4 ± 9.9	57.9 ± 11.2	100U BTX-A + hydrogel	normal saline + hydrogel	57/58	3
5^*^	^2^ Chermansky et al., 2022	United States of America	2017–2020	OAB	RCT	294	107/11	59.1 ± 11.3	57.9 ± 11.2	300U BTX-A + hydrogel	normal saline + hydrogel	60/58	3
5^**^	^3^ Chermansky et al., 2022	United States of America	2017–2020	OAB	RCT	294	107/11	59.8 ± 9.1	57.9 ± 11.2	400U BTX-A + hydrogel	normal saline + hydrogel	60/58	3
5^***^	^4^ Chermansky et al., 2022	United States of America	2017–2020	OAB	RCT	294	107/10	60.7 ± 9.4	57.9 ± 11.2	500U BTX-A + hydrogel	normal saline + hydrogel	59/58	3

Range: () = range; mean ± SD; OAB, overactive bladder; IC/BPS, interstitial cystitis/bladder painful syndrome; BTX-A, botulinum toxin A; Pb, placebo; Tx, treatment.

### Quality assessment

The Cochrane Risk of Bias Tool was utilized to assess the quality of the articles. The evaluation criteria included the following items: random sequence generation, allocation concealment, blinding (of participants, therapists, and outcome assessors), incomplete outcome data, selective outcome reporting, and other sources of bias. Each item was categorized as high risk, low risk, or unclear risk. Ultimately, the overall quality of the articles was classified into three categories: (1) low risk of bias, (2) high risk of bias, and (3) unknown risk of bias.

### Statistical analysis

The weighted mean difference (WMD) and 95% confidence interval (CI) was were used to describe continuous outcomes. The odds ratio (OR) with a 95% CI was used to evaluate the binary categorical variables. For data that conformed to a normal distribution, the mean and standard deviation (SD) were transformed (Wan et al., 2014; Luo et al., 2018). In addition, for some studies that provided only the initial and final results and their SDs, the corresponding effect sizes were calculated using the following Equations 1, 2. To assess the stability of the results, R was set to 0.5 and its value was adjusted Higgins JPTTJ, Chandler J, Cumpston M, Li T, Page MJ, Welch VA. Cochrane Handbook for Systematic Reviews of Interventions Version 6.2 (Updated February 2021). Cochrane; 2021.

$$\Delta X=\left|X1‐X2\right|$$ (1)

$$\Delta S*\;\Delta S=S1*S1+S2*S2-2*R*S1*S2\;\left(R\text{ is }a\text{ constant}\right)$$ (2)

The level of the heterogeneity was assessed using the Cochrane Q test and I^2^ statistics. Heterogeneity was indicated when I^2^ > 50% or when the p-value for the Q statistic was <0.05. The statistical significance was defined as a two-sided P-value <0.05. Random effects models were used to estimate the size of the collective effect and minimize potential bias. Sensitivity analysis was conducted to evaluate the robustness of the overall effect estimates. Subgroup analyses were performed based on various follow-up times and doses of BTX-A. Statistical analyses were conducted using Review Manager (RevMan version 5.3, the Nordic Cochrane Center, the Cochrane Collaboration, 2014) and Stata software (version 16; Stata Corp LLC, College Station, TX, United States).

## Results

### Leatures searched and quality assessment

**FIGURE 1:**
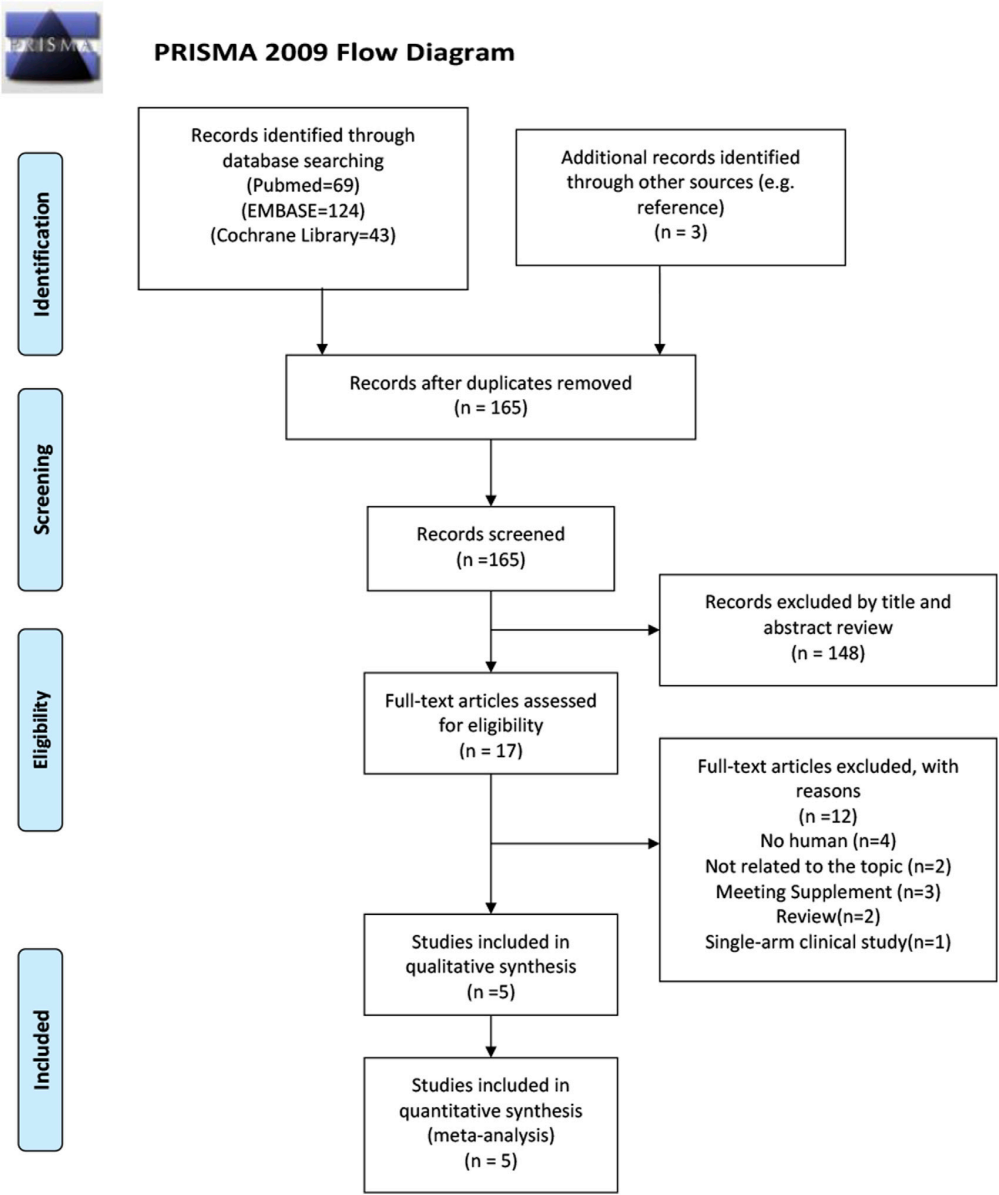
PRISMA flow diagram of literature retrieval. PRISMA, Preferred Reporting Items for Systematic Reviews and Meta-Analyses.

Following the search strategy, a total of 239 articles were initially identified, including 69 citations from PubMed, 124 citations from EMBASE, 43 citations from the Cochrane Library, and 3 additional records from references. 74 articles were removed due to duplication. Titles and abstracts were reviewed, and 148 articles unrelated to the topic were excluded. After reviewing the full texts, 12 articles were excluded for the following reasons: 4 studies involved non-human subjects, 2 studies were unrelated to the topic, 3 studies were meeting supplements, 2 studies were reviews, and 1 study was a single-arm clinical trial. Ultimately, a total of 11 trials from 5 studies involving 500 patients (357 in the experimental group and 143 in the control group) were included. The flowchart of the literature screening is presented in Figure 1. The quality assessment of the included research is shown in Supplementary Figure S1. The overall risk of bias in these articles was low.

### Meta-analysis of intravesical instillation between BTX-A and placebo group for IC/BPS or OAB

#### Effect of bladder instillation of BTX-A on outcomes

**FIGURE 2:**
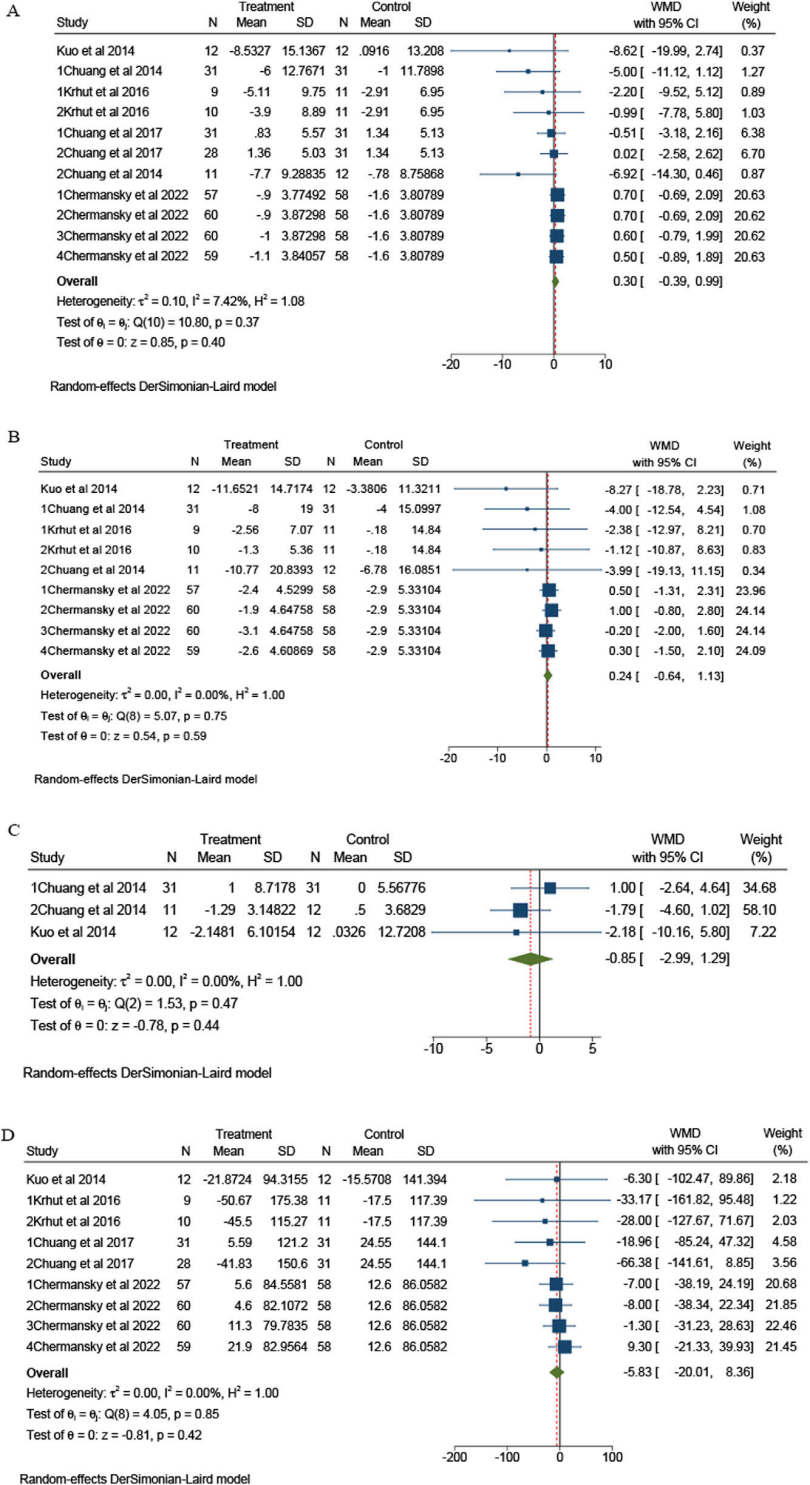
The forest plots of change of urinary symptoms for the BTX-A intravesical instillation compared to the placebo. **(A)** Frequency per 3 days; **(B)** Urinary urgency per 3 days; **(C)** Urgency urinary incontinence; **(D)** Void volume. CI, confidence interval.

No statistically significant differences were observed in the meta-analysis study. In terms of major efficacy outcomes, 11 trials from 5 studies reported on the frequency of episodes (Chuang et al., 2014; Kuo et al., 2014; Krhut et al., 2016; Chuang and Kuo, 2017; Chermansky et al., 2022). Meta-analysis showed no statistically significant difference in urinary frequency episodes per 3 days between intravesical instillation of BTX-A and placebo group (overall WMD = 0.3; 95%CI: -0.39 to 0.99, *p* = 0.37) and without heterogeneity (I^2^ = 7.42%), as presented in Figure 2A. There are nine trials from four studies that reported episodes of urinary urgency (Chuang et al., 2014; Kuo et al., 2014; Krhut et al., 2016; Chermansky et al., 2022). The meta-analysis revealed no statistically significant difference in urinary urgency episodes over a 3-day period between the intravesical instillation of BTX-A and the placebo group (overall WMD = 0.24; 95%CI: -0.64 to 1.13, *p* = 0.75), with no observed heterogeneity (I^2^ = 0%), as illustrated in Figure 2B. There are three trials from two studies that reported UUI (Chuang et al., 2014; Kuo et al., 2014). The meta-analysis indicated no statistically significant difference in UUI between the intravesical instillation of BTX-A and the placebo group (overall WMD = −0.85; 95%CI: -2.99 to 1.29, *p* = 0.47), also showing no heterogeneity (I^2^ = 0%), as depicted in Figure 2C.

There are nine trials from four studies that reported on VV (Kuo et al., 2014; Krhut et al., 2016; Chuang and Kuo, 2017; Chermansky et al., 2022). The meta-analysis revealed no statistically significant difference in UUI between the intravesical instillation of BTX-A and the placebo group (overall WMD = −5.83; 95%CI: -20.01 to 8.36, *p* = 0.85), with no observed heterogeneity (I^2^ = 0%), as illustrated in Figure 2D.

**FIGURE 3:**
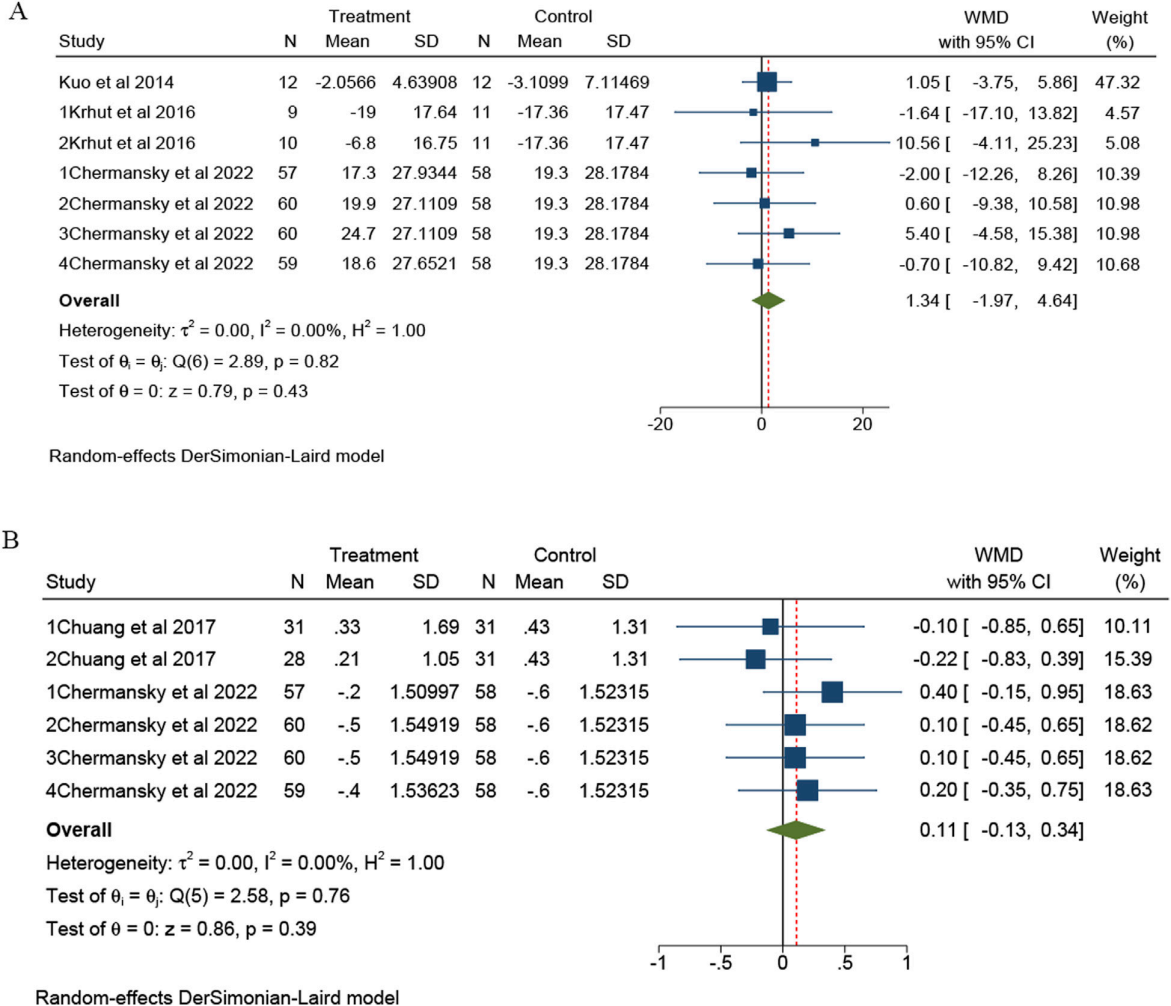
The forest plots of change of OABSS **(A)** and nucturia **(B)** for BTX-A intravesical instillation compared to the placebo. OABSS, overactive bladder syndrome score.

There are seven trials from three studies that reported the OABSS (Kuo et al., 2014; Krhut et al., 2016; Chermansky et al., 2022). The meta-analysis revealed no statistically significant difference in OABSS between the intravesical instillation of BTX-A and the placebo group (overall WMD = 1.34; 95% CI: -1.97 to 4.64, *p* = 0.82), with no observed heterogeneity (I^2^ = 0%), as illustrated in Figure 3A. Additionally, six trials from two studies reported nocturia (Chuang and Kuo, 2017; Chermansky et al., 2022). The meta-analysis indicated no statistically significant difference in nocturia between the intravesical instillation of BTX-A and the placebo group (overall WMD = 0.11; 95%CI: -0.13 to 0.34, *p* = 0.76), also showing no heterogeneity (I^2^ = 0%), as depicted in Figure 3B.

**FIGURE 4:**
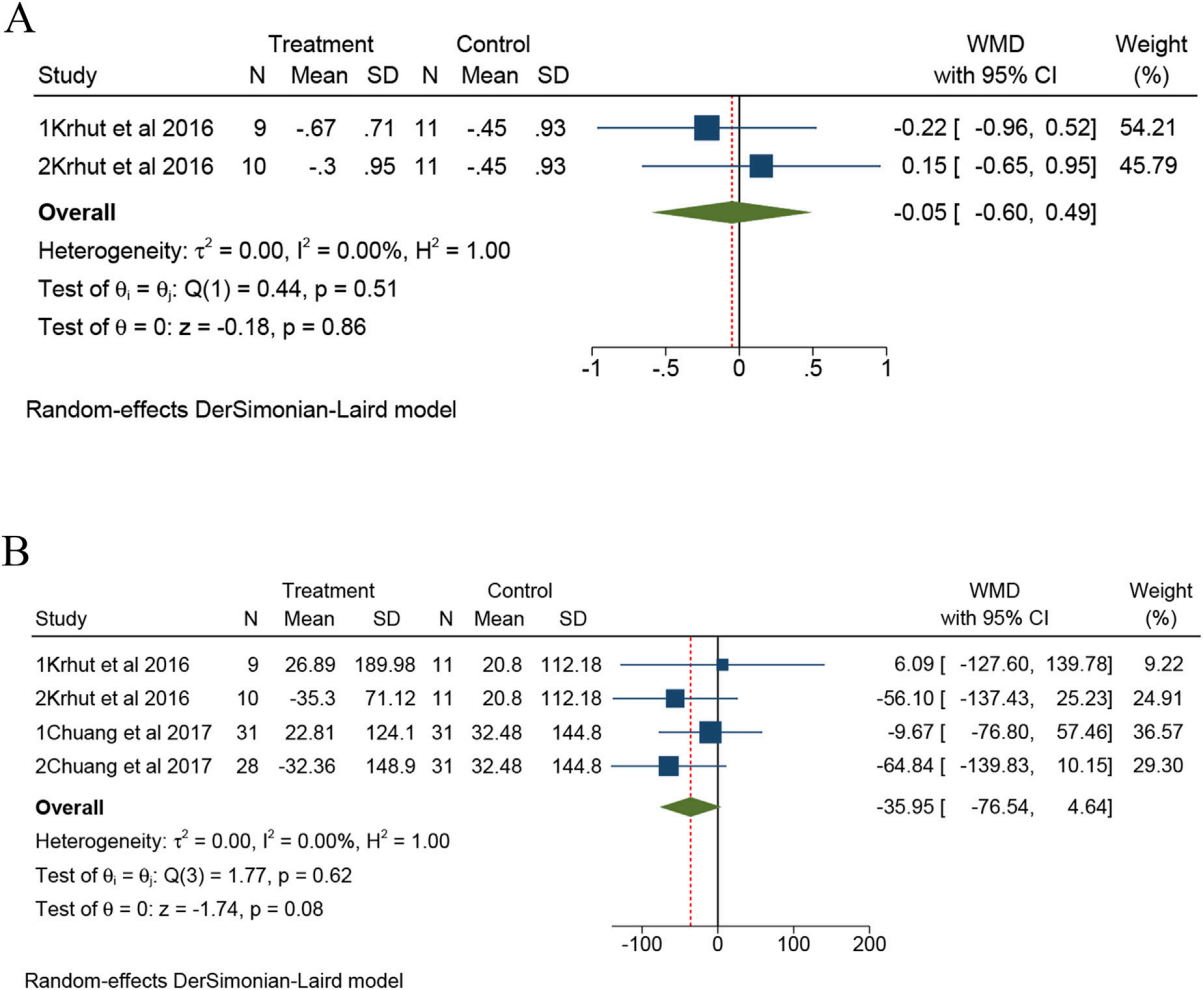
The forest plots of change of PPBC **(A)** and Cystometric capacity **(B)** for BTX-A intravesical instillation compared to the placebo. PPBC, Patient Perception of Bladder Condition.

There are two trials from one study that reported PPBC (Krhut et al., 2016). Meta-analysis showed no statistically significant difference in PPBC between intravesical instillation of BTX-A and placebo group (overall WMD = −0.05; 95%CI: -0.60 to 0.49, *p* = 0.51) and without heterogeneity (I^2^ = 0%), as presented in Figure 4A.

There are four trials from two studies that reported cystometric capacity (Krhut et al., 2016; Chuang and Kuo, 2017). Meta-analysis showed no statistically significant difference in cystometric capacity (overall WMD = −35.95; 95%CI: -76.54 to 4.64, *p* = 0.62). Compared to the placebo group, the intravesical instillation of BTX-A did not significantly increase cystometric capacity. There was no observed heterogeneity (I^2^ = 0%). All results are presented in Figure 4B.

### Safety of bladder instillation of BTX-A on outcomes

**FIGURE 5:**
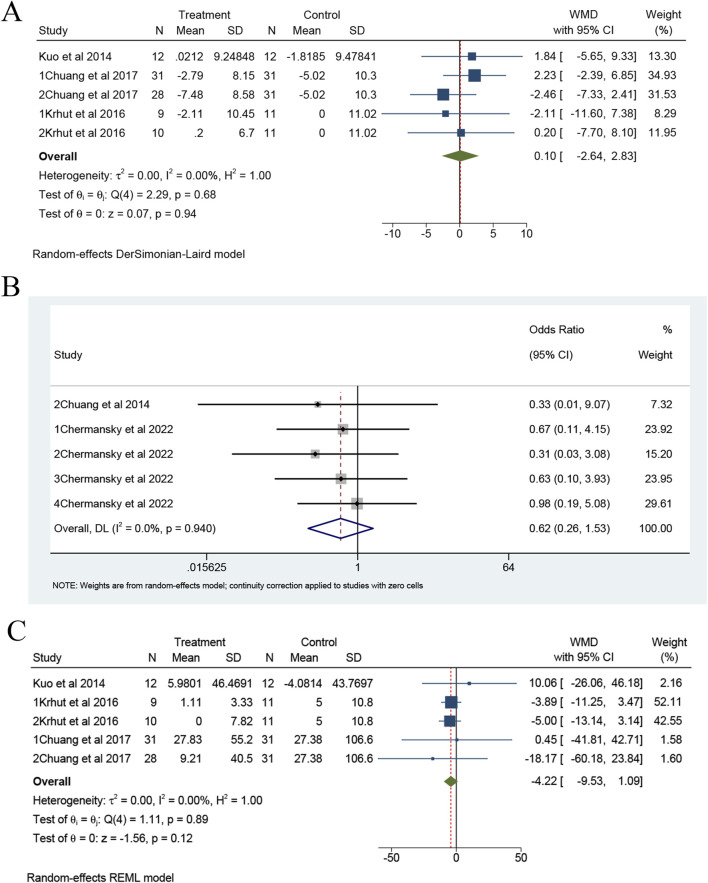
The forest plots of change of Q_max_ **(A)**, UTI **(B)** and PVR **(C)** for BTX-A intravesical instillation compared to the placebo. UTI, urinary tract infection; PVR: post-void residual.

In terms of minor safety outcomes, both Q_max_ and UTI events were evaluated. There are five trials from three studies that reported Q_max_ (Kuo et al., 2014; Krhut et al., 2016; Chuang and Kuo, 2017). Meta-analysis showed no statistically significant difference in Q_max_ between intravesical instillation of BTX-A and placebo group (overall WMD = 0.10; 95%CI: -2.64 to 2.83, *p* = 0.68), with no observed heterogeneity (I^2^ = 0%), as illustrated in Figure 5A. Additionally, five trials from two studies reported UTI events, shown in Figure 5B (Chuang et al., 2014; Chermansky et al., 2022). As shown in Figure 5C, the forest plots of change of PVR showed no significant difference between the two groups (*p* = 0.89).

### The subgroup analysis of intravesical instillation of BTX-A and normal saline for IC/BPS or OAB

In the present study, subgroup analyses were performed after stratification for different follow-up durations (4 W vs. 12 W) and BTX-A infusion doses (≤200 U vs. > 200 U). However, in subgroup analyses, no statistically significant differences were detected between adjusted intravesical instillation of BTX-A and placebo group (Supplementary Figures S2, 3). Interestingly, it was found that adjusted follow-up time and BTX-A dosage affected VV.

Among them, the increased of VV at 4 weeks of follow-up (WMD = −31.99, 95%CI: -70.53 to 6.54) was significant observed compared to that at 12 W (WMD = −1.73, 95%CI: -16.98 to 13.53), the between-groups difference in outcome (pooled WMD = −5.83, 95%CI: -20.01 to 8.36) and without heterogeneity (I^2^ = 0%). In contrast to the >200 U BTX-A groups (WMD = −0.07, 95%CI: -17.56 to 17.42), patients in the ≤200 U botulinum toxin group (WMD = −16.89, 95%CI: -41.14 to 7.35) had a markedly increased VV even though the p-value was not statistically significant. The results of between-groups (pooled WMD = −5.83, 95%CI: -20.01 to 8.36) and without heterogeneity (I^2^ = 0%).

### Sensitivity analysis

Sensitivity analysis was conducted by omitting individual studies sequentially. As shown in Supplementary Figure S4, the aggregated ORs of the remaining studies did not exceed the estimated range, according to the meta-analysis of each group. Moreover, there were no significant differences between the adjusted and preliminary aggregated estimates, showing that our meta-analysis was robust.

### Publication bias

Cystometric capacity, nocturia, OABSS, void volume, UUI, Q_max_, UTI or PVR were showed no evidence of publication bias.

## Discussion

In this study, a total of 11 trials from 5 studies, involving 500 patients (357 in the experimental group and 143 in the control group) were included. Despite conducting a meta-analysis, no statistically significant findings were observed across all outcome variables. Nevertheless, for UUI and VV, the results, while not statistically significant, generally favored the use of BTX-A bladder instillation. Regarding safety, intravesical instillation of BTX-A demonstrated a reduced risk of UTI and PVR compared to the placebo group, as illustrated in Figure 5.

BTX-A injections have been widely used for the treatment of OAB and IC/BPS. However, the side effects and discomfort associated with repeated injections may lead some patients to discontinue this therapeutic approach (Cui et al., 2013). Consequently, the utilization of novel non-invasive instillation modalities of BTX-A is imperative for the management of patients. Given its molecular weight of 150 kDa, BTX-A, a large protein, encounters challenges in penetrating the urinary epithelium following bladder instillation (Soler et al., 2008; Donget al., 2019). This difficulty is primarily attributed to the presence of tight junctions within the urinary epithelium. Therefore, both animal experiments and clinical trials are essential to confirm the viability of its instillation.

Chuang et al. conducted an animal study to investigate the efficacy of bladder instillation of BTX-A in mitigating the inflammatory response and bladder hypersensitivity induced by cyclophosphamide (Chuang et al., 2009b). The findings indicate that the intravesical instillation of BTX-A mitigates the bladder inflammation and hypersensitivity induced by cyclophosphamide. Cystometrograms demonstrated a significant reduction in both intercontraction interval (ICI) (a 107% decrease) and contraction amplitude (a 43% decrease) in the group treated with BTX-A compared to the placebo group. Krhut et al. also conducted animal experiments and preliminary clinical trials (Krhut and Zvara, 2011). The researchers assessed the efficacy of BTX-A instillation before and after instillation in 16 patients with refractory OAB. The study revealed that intravesical administration of BTX-A yielded comparable cystometrograms alterations to those observed with BTX-A instillation into the bladder. Furthermore, the clinical investigation indicated that patients treated with BTX-A experienced symptom amelioration and a decrease in the average frequency of urinary incontinence episodes (Krhut and Zvara, 2011).

Subsequently, Chuang et al. assessed the feasibility of using liposomes for the encapsulation of BTX-A for bladder instillation (Chuang et al., 2009a). On the first day, bladder instillation was conducted in rats using liposomes and BTX-A separately or in combination. Seven days later, bladder instillation was carried out with acetic acid. The findings indicated that the group treated with liposome-encapsulated BTX-A exhibited a significant reduction in the changes in ICI induced by acetic acid instillation. The findings indicated that the group treated with liposome-encapsulated BTX-A demonstrated a notable reduction in the alterations in ICI caused by acetic acid infusion, as well as in the expression levels of calcitonin gene-related peptide and synaptosomal-associated protein 25.

The efficacy of noninvasive BTX-A bladder instillation in animal studies has facilitated advancements in clinical trials. Kuo et al. (Kuo et al., 2014) conducted a single-center, double-blind, randomized controlled clinical trial in which they recruited 24 OAB patients. These participants were randomly assigned to either a normal saline-perfused group or a liposome-encapsulated BTX-A-perfused group (80 mg liposomes and 200 U BTX-A). The results showed that the group treated with liposome-encapsulated BTX-A experienced a significant reduction in urinary frequency (*p* = 0.008) and the frequency of urgency episodes (*p* = 0.012) without a substantial elevation in the likelihood of PVR and UTI. In a multicenter prospective randomized controlled trial, Chuang et al. (Chuang et al., 2014) evaluated the efficacy of liposome-encapsulated BTX-A in treating OAB. The findings indicated that this treatment effectively alleviates the clinical symptoms of OAB, without adverse effects. Thus, liposome-encapsulated BTX-A shows considerable potential as a future treatment option for OAB. Krhut et al. (Krhut et al., 2016) conducted a novel BTX-A bladder instillation method by combining BTX-A with TC-3 gel, a temperature-sensitive hydrogel, followed by bladder instillation (200 U of BTX-A and 50 mL of TC-3 gel). The study results indicated that the combination of BTX-A and TC-3 gel significantly enhanced the frequency of urgency episodes, leakage episodes, as well as the total scores on the Overactive Bladder Questionnaire and Patient Perception of Bladder Condition, compared to other bladder instillation methods such as saline, DMSO, and DMSO combined with BTX-A instillation (Krhut et al., 2016). Chermansky et al. (Chermansky et al., 2022) conducted a similar clinical trial involving the use of hydrogel-coated BTX-A bladder instillation; however, their results diverged from previous findings. By enrolling 607 patients who were randomly assigned to the placebo group, 100 U BTX-A + hydrogel group, 300 U BTX-A + hydrogel group, 400 U BTX-A + hydrogel group, and 500 U BTX-A + hydrogel group. The results showed that, compared to the placebo group, there were no statistically significant alterations in the frequency of urgency episodes, frequency of voiding episodes, and other outcome measures across the various concentrations of BTX-A bladder instillations. No significant changes were noted in the placebo group either. The validity of animal experiments has not been convincingly demonstrated in clinical trials, especially regarding the ongoing controversy about the efficacy of treatment following hydrogel-assisted BTX-A bladder instillation. Additionally, the limited long-term effectiveness of liposomal BTX-A raises questions about its potential for BTX-A instillation in therapeutic applications. Therefore, pooling clinical studies to evaluate the overall treatment outcomes may provide a clearer understanding of the feasibility of bladder instillation of BTX-A for the treatment of OAB or IC/BPS.

In the management of IC/BPS, relevant clinical trials have demonstrated the feasibility of bladder instillation with BTX-A encapsulated in liposomes or hydrogels. Nevertheless, the variability of the outcome variable may be influenced by a potentially insufficient sample size, leading to variability in the results. Therefore, large sample sizes are essential to assess the viability of using liposome- or hydrogel-encapsulated BTX-A for intravesical instillation. Although some clinical trials have not yielded statistically significant results, they have still indicated an improvement in symptoms compared to baseline levels, as well as the feasibility of the treatment. Regarding the continued efficacy of BTX-A in the treatment of other types of lower urinary tract dysfunction, He et al. conducted a meta-study of BTX-A in the treatment of neurogenic bladder in children, and the results of 19 included studies showed a mean increase in maximal bladder capacity of 97.7 mL after BTX-A injections (34.1%–162% increase), and a decrease in maximal urethral pressure of cm H_2_O, and bladder compliance increased by 5.3 mL/cm H_2_O (He et al., 2024). These results strongly suggest that intramuscular injection of BTX-A into the urethra enhances bladder capacity, compliance, and maximal neurogenic urethral overactivity. In addition, lower urinary tract dysfunction often causes bladder fibrosis. Feng et al. showed that mechanosensitive Piezo1 channels are involved in the progression of neurogenic bladder fibrosis and pro-fibrotic alterations in SV-HUC-1 cells, and this pathway also provides insight into the mechanism of botulinum toxin A in treating fibrosis altered by lower urinary tract dysfunction (Feng et al., 2024).

This study has certain limitations. Firstly, due to the restricted availability of literature on bladder instillation with BTX-A, our meta-analysis was constrained to a limited number of articles. Consequently, the transformation of data from some of these articles may have influenced the overall outcome and the limitations of the valid research literature may cause some bias in the results. Secondly, heterogeneity was observed across studies in the selection of placebos for the control group. Furthermore, there was heterogeneity in the selection of diseases for the overall outcome goal, encompassing OAB and IC/BPS. Nevertheless, the primary objective was able to demonstrate the efficacy of BTX-A instillation.

## Conclusion

The utilization of bladder instillation of BTX-A for the management of OAB or IC/BPS remains a topic of debate. Further research is required to confirm the viability and safety of various methods of administering BTX-A into the bladder. This study illustrated that while the overall outcome metrics did not reach statistical significance, there was evidence of the promising effectiveness of beneficial bladder instillation of botulinum toxin for certain metrics.

## Data Availability

The original contributions presented in the study are included in the article/Supplementary Material, further inquiries can be directed to the corresponding author.

## Supplementary material

SUPPLEMENTARY FIGURE S1 Detailed quality assessment of included RCTs. **(A)** Risk of bias graph. **(B)** Risk of bias summary.

SUPPLEMENTARY FIGURE S2 The subgroup analysis of change of indicators for BTX-A intravesical instillation compared to the placebo. **(A)** Subgroup analysis of follow-up time for frequency; **(B)** Subgroup analysis of drug dose of BTX-A for frequency; **(C)** Subgroup analysis of follow-up time for nucturia; **(D)** Subgroup analysis of drug dose of BTX-A for nocturia; **(E)** Subgroup analysis of follow-up time for OABSS; **(F)** Subgroup analysis of drug dose of BTX-A for OABSS. OABSS, overactive bladder syndrome score.

SUPPLEMENTARY FIGURE S3 The subgroup analysis of change of indicators for BTX-A intravesical instillation compared to the placebo. **(A)** Subgroup analysis of follow-up time for urinary urgency; **(B)** Subgroup analysis of drug dose of BTX-A for urinary urgency; **(C)** Subgroup analysis of follow-up time for void volume; **(D)** Subgroup analysis of drug dose of BTX-A for void volume.

SUPPLEMENTARY FIGURE S4 Sensitivity analyses of outcomes. **(A)** Cystometric capacity; **(B)** Frequency; **(C)** Nocturia; **(D)** OABSS; **(E)** Post-Void Residual; **(F)** Q_max_; **(G)** Urinary urgency; **(H)** Urinary Tract Infection; **(I)** Urgency urinary incontinence; **(J)** Void volume; CI, confidence interval.
