# Supplementary figures and images for: Safety and efficacy of intravesical instillation of botulinum toxin-A in the treatment of interstitial cystitis/bladder pain syndrome and overactive bladder: a systematic review and meta-analysis
Source: Front Pharmacol. 2025 Apr 15;16:1586845. doi: 10.3389/fphar.2025.1586845 (PMC12037478; doi:10.3389/fphar.2025.1586845)

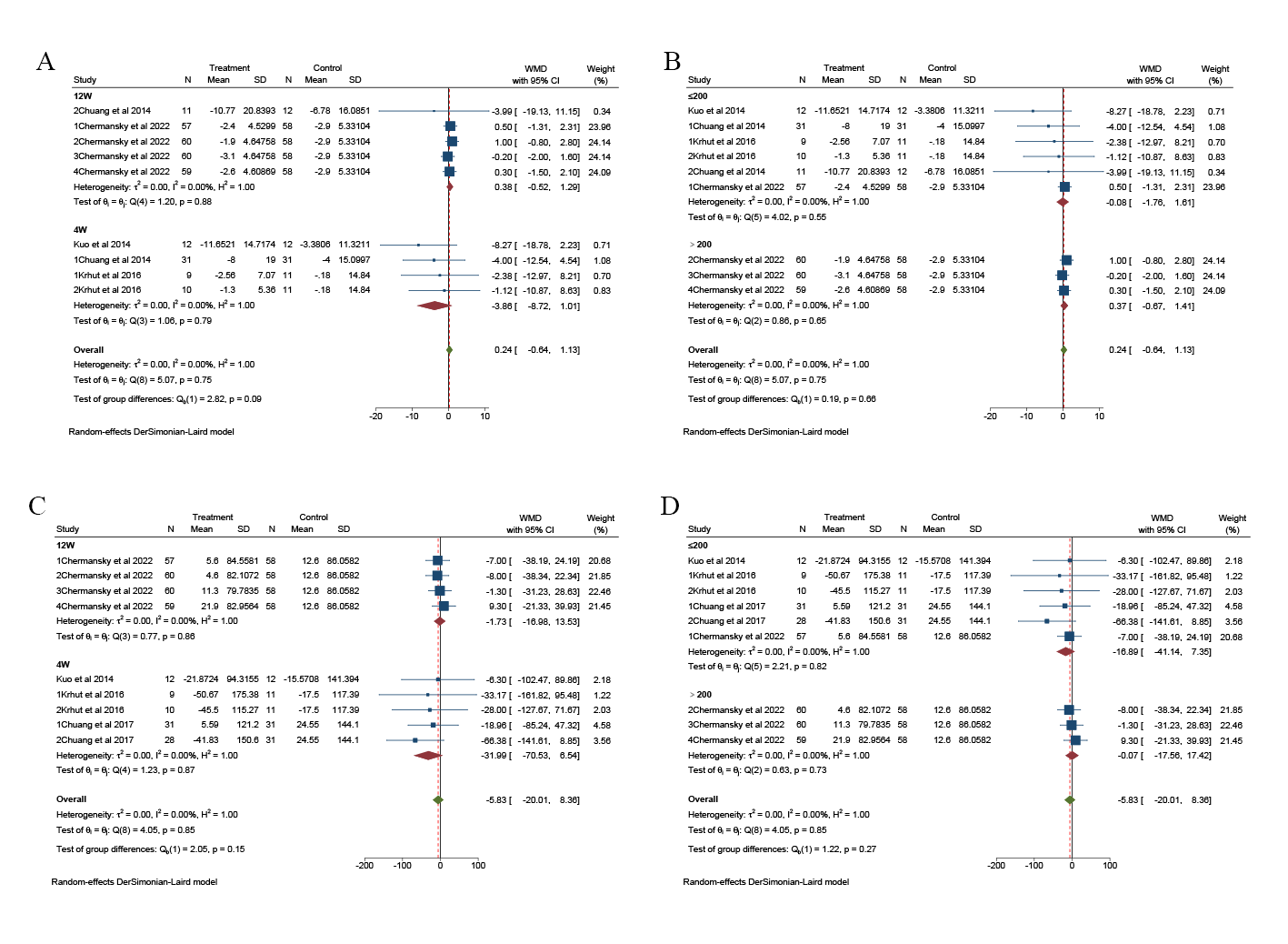

Supplement: Supplementary file 1 [file Image3.tif]

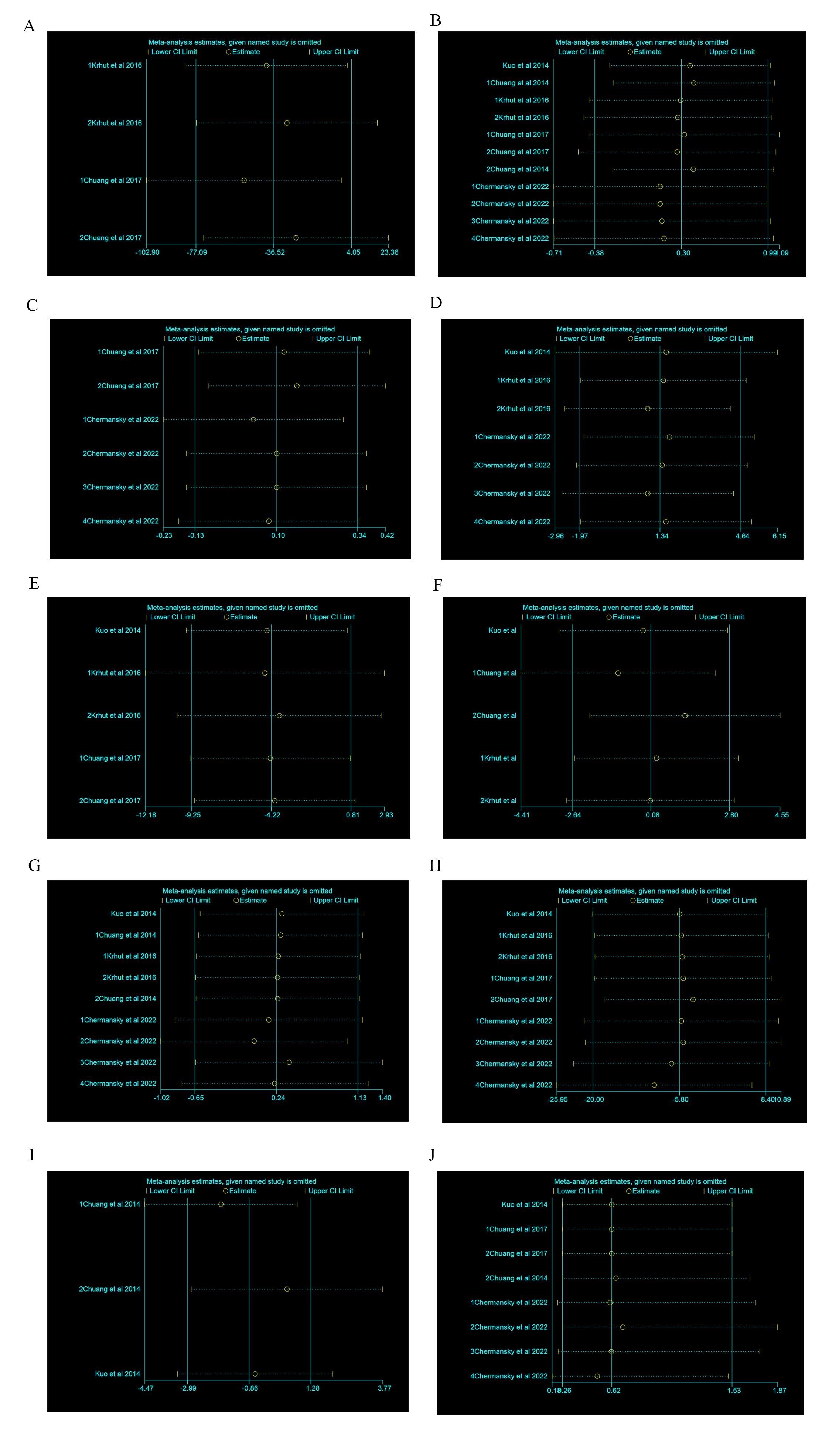

Supplement: Supplementary file 2 [file Image4.tif]

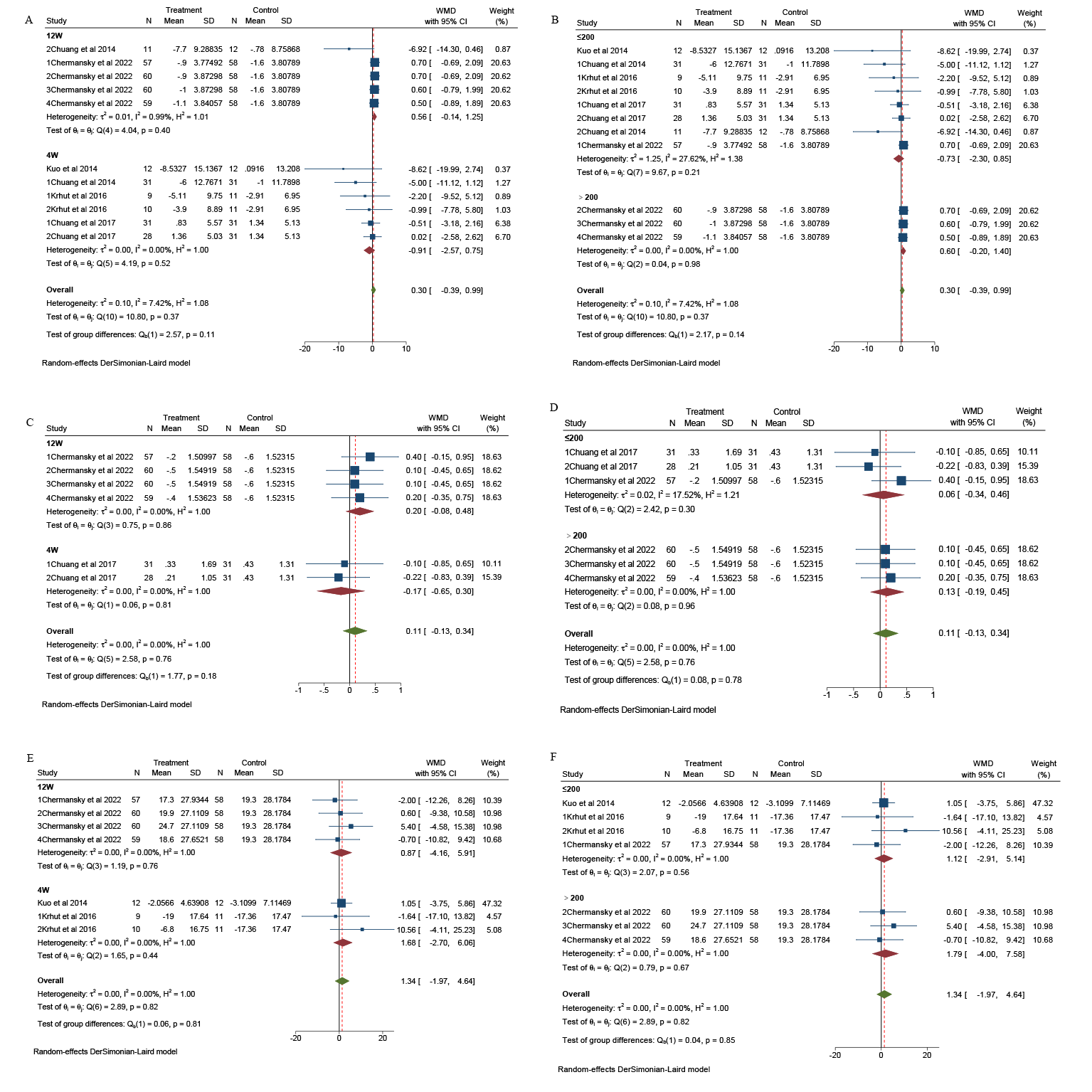

Supplement: Supplementary file 3 [file Image2.tif]

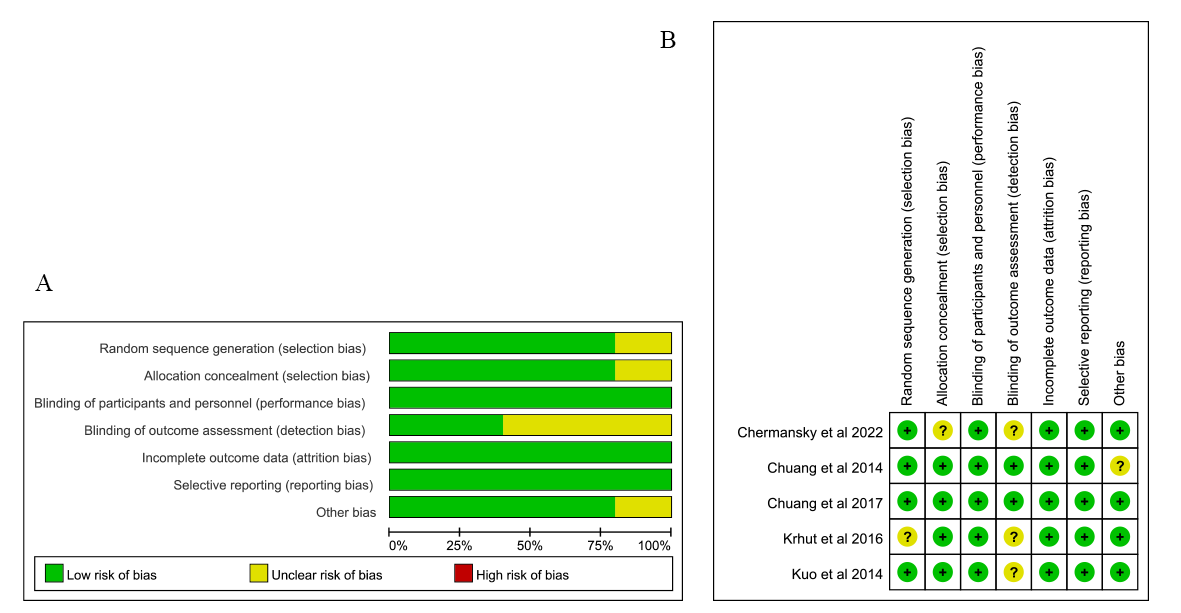

Supplement: Supplementary file 4 [file Image1.tif]
